# The Effect of Silver Nanoparticles Size, Produced Using Plant Extract from *Arbutus unedo*, on Their Antibacterial Efficacy

**DOI:** 10.3390/nano7070178

**Published:** 2017-07-10

**Authors:** Nicholas Skandalis, Anastasia Dimopoulou, Anthie Georgopoulou, Nikolaos Gallios, Dimitrios Papadopoulos, Dimitrios Tsipas, Ioannis Theologidis, Nikolaos Michailidis, Maria Chatzinikolaidou

**Affiliations:** 1 Department of Phytopathology, Benaki Phytopathological Institute, 8 St. Delta, Kifissia, GR-14561 Athens, Greece; n.skandalis@bpi.gr (N.S.); anastasia_dimopoulou@imbb.forth.gr (A.D.); i.theologidis@bpi.gr (I.T.); 2 Department of Materials Science and Technology, University of Crete, 71001 Heraklion, Greece; anthieg87@yahoo.com (A.G.); mchatzin@materials.uoc.gr (M.C.); 3 Department of Mechanical Engineering, Aristotle University of Thessaloniki, 54124 Thessaloniki, Greece; gallios@auth.gr (N.G.); dhpapado@auth.gr (D.P.); dtsipas@auth.gr (D.T.); 4 Institute of Electronic Structure and Laser (IESL), Foundation for Research and Technology Hellas (FORTH), N. Plastira 100, 70013 Heraklion, Greece

**Keywords:** green synthesis, silver nanoparticles, particle size, antibacterial efficacy

## Abstract

Silver nanoparticles (AgNPs) have been demonstrated to restrain bacterial growth, while maintaining minimal risk in development of bacterial resistance and human cell toxicity that conventional silver compounds exhibit. Several physical and chemical methods have been reported to synthesize AgNPs. However, these methods are expensive and involve heavy chemical reduction agents. An alternative approach to produce AgNPs in a cost-effective and environmentally friendly way employs a biological pathway using various plant extracts to reduce metal ions. The size control issue, and the stability of nanoparticles, remain some of the latest challenges in such methods. In this study, we used two different concentrations of fresh leaf extract of the plant *Arbutus unedo* (LEA) as a reducing and stabilizing agent to produce two size variations of AgNPs. UV-Vis spectroscopy, Dynamic Light Scattering, Transmission Electron Microscopy, and zeta potential were applied for the characterization of AgNPs. Both AgNP variations were evaluated for their antibacterial efficacy against the gram-negative species *Escherichia coli* and *Pseudomonas aeruginosa*, as well as the gram-positive species *Bacillus subtilis* and *Staphylococcus epidermidis*. Although significant differences have been achieved in the nanoparticles’ size by varying the plant extract concentration during synthesis, the antibacterial effect was almost the same.

## 1. Introduction

AgNPs have emerged as a new class of antimicrobials with potential against a range of pathogens [1,2], including those that have developed antibiotic resistance [3,4]. In addition, their implementation in aseptic surgical and procedural techniques could prevent infections that require medical apparatus removal, i.e., implants, and long term systemic antibiotic therapy, thus reducing healthcare costs [5]. 

*Staphylococcus epidermidis*, a natural inhabitant of human skin, is a prominent member of coagulase negative staphylococci, the most commonly isolated pathogens from infection sites around blood or orthopedic apparatus [6]. The success of *S. epidermidis* as a pathogen is attributed to its ability to adhere to diverse surfaces, including a range of biomaterials, where it forms a biofilm [7,8]. *Pseudomonas aeruginosa* is the most prevalent respiratory pathogen in immunocompromised patients [9,10], with an innate capability to develop multi-drug resistance due to efflux pumps that expel antibiotics and other noxious molecules [11]. A similar apparatus is also found in *Escherichia coli*. 

The phyto-mediated synthesis of silver nanoparticles (AgNPs) is a rapid, cost-effective, and easily scalable-up synthetic route. Various leaf extracts from plants like *Lantana camara* L. [12], *Protium serratum* [13], and *Eriobotrya japonica* [14] have been reported to be very effective in reducing silver salts to AgNPs. Based on a previous work of coauthors, presenting the synthesis of AgNPs produced using the leaf extract of the plant *Arbutus unedo* (LEA) as a reducing and stabilizing agent [15], we aim to extend the advantages of this low-cost and environmentally friendly method for the production of AgNPs. We also present here the development of two new products, silver nanoparticles variation 1 (NP1) and variation 2 (NP2), which are produced by varying the amount of LEA in the synthesis and leading to different size and size distribution of the products. For the first time, we investigate the antibacterial efficacy of NP1 and NP2, applying them against four bacterial strains, which are relevant for biomedical applications. We aim to gain a better insight into the complex action of AgNPs on bacterial cells, and with this perspective, to incorporate them into antibacterial biomaterial scaffolds for tissue regeneration. Furthermore, we report on the characterization of the two variations of AgNPs by means of Ultraviolet-Visible spectroscopy (UV-Vis), Dynamic Light Scattering (DLS), Transmission Electron Microscopy (TEM), and zeta potential. Moreover, we evaluate the antibacterial efficacy by applying different concentrations of both AgNPs variations against the gram-negative species *E. coli* and *P. aeruginosa*, as well as the gram-positive species *Bacillus subtilis* and *S. epidermidis*. By means of Scanning Electron Microscopy (SEM), we visualize the morphological changes of *E. coli* membranes under the induction of both AgNP variations.

## 2. Materials and Methods

### 2.1. Production and Characterization of Green Chemistry Silver Nanoparticles

Fresh *Arbutus unedo* leaves were collected from the surroundings of Thessaloniki region, Macedonia, Greece. The fresh leaf extract (LEA) used for the reduction of Ag^+^ ions to Ag° was prepared by placing 10 g of thoroughly washed finely cut leaves in 500 mL flask, along with 100 mL of distilled water, and then boiling the mixture for 15 min before decanting it. The LEA was centrifuged at 5000 rpm for 15 min to remove impurities, by collecting only the supernatant, having a pH of 4.1. The concentration of phenolic compounds and flavonoids in the LEA for *A. unedo* is quite considerable [16,17]. Both are considered to be responsible for the reducing and stabilizing action of the LEA.

Two AgNPs variations were prepared, differing in the quantity of LEA used in their synthesis. An aqueous (distilled water) solution of 3 mM AgNO_3_ was prepared at room temperature and mixed with the LEA, while stirring magnetically at 1000 rpm for 30 s at a temperature of 80 °C. In this context, 1 volume of LEA was mixed with 10 volumes of 3 mM AgNO_3_ to make NP2, while 2 volumes of LEA were mixed with 10 volumes of 3 mM AgNO_3_ to make NP1 solution. A total of 100 mL solution (after mixing) was prepared in both cases, with the pH ranging close to 4.3.

#### 2.1.1. UV-Vis Spectroscopy

The optical properties were analyzed using UV-Vis spectroscopy (SHIMADZU CORPORATION, Tokyo, Japan) by monitoring the electron spectra of the samples employing a Shimadzu UV-1800 UV-Vis spectrophotometer. The spectral bandwidth ranged from 190 to 1100 nm at a wavelength resolution of 1 nm, while quartz cuvettes were applied for the measurements, over a path-length of 10 mm. The device was fully controlled by UV Probe software package.

#### 2.1.2. Dynamic Light Scattering

A VASCO DLS device (Cordouan Technologies, Pessac, France) was used to measure the hydrodynamic diameter of both AgNPs variations. The hydrodynamic diameter showed the particle size distribution of the colloidal AgNPs variations and provided knowledge about the particle dispersion. NanoQ software package facilitated controlling of hardware and analyzing of results.

#### 2.1.3. Transmission Electron Microscopy

Dispersions of both nanoparticle variations were sonicated for 20 min and a drop of each diluted sample, adjusted to a concentration of 75 μg/mL, was placed on a carbon grid and allowed to dry overnight. The next day samples were observed using a JEOL JEM-2100 (JEOL, Peabody, MA, USA) high-resolution transmission electron microscope.

#### 2.1.4. Zeta Potential

Dispersions of both nanoparticle variations (NP1 and NP2) were sonicated for 20 min and diluted to final concentration of 75 μg/mL in phosphate buffer saline (PBS) pH = 7.4. The zeta potential of either pure nanoparticle variations NP1 and NP2 and *E. coli* cells were measured using a Zetasizer Nano ZS instrument (Malvern, Worcestershire, UK).

### 2.2. Efficacy Evaluation Assays

#### 2.2.1. Bacterial Strains and Growth Conditions

Bacterial strains included *S. epidermidis* isolate C5M6 (ACA-DC 4057; Agricultural University of Athens Dairy Bacteria Collection), *B. subtilis* subsp. *subtilis* isolate BgG11 (ACA-DC 4045), *E. coli* DH5a, and *P. aeruginosa* ADD1976. Strains were routinely grown at 28 °C in Luria-Bertani (LB) broth or on LB agar medium. 

#### 2.2.2. Broth Microdilution Method

Overnight grown stationary phase cultures of each bacterial strain were streaked on LB plates to check their purity. Cultures were then adjusted to a concentration of 5 × 10^6^ cfu mL^−1^. The adjusted bacterial *inocula* (10 μL) were added to each well of sterile U based microtitre plates (COSTAR 3595, Corning Incorporated, Corning, NY, USA) containing appropriate concentration of LB medium and NPs to reach 1× and test concentrations, respectively in the total volume, which was 100 μL on each well. Consequently, a final inoculum concentration of 105 cfu mL^–1^ was obtained in each well.

One target bacterium was assayed on each plate, representing one experiment, in quadruplicates (wells) for each of the 75, 30, 15, 3, 0.3, or 0 (control) μg/mL NP concentrations. Blank wells (NPs and medium only) containing each test concentration were also included in triplicates. Three independent biological experiments (*inocula*, NPs, tested plates) were performed for each target bacterium. Growth kinetics were monitored for 48 h at 37 °C based on optical density (OD). OD was measured using triplicate readings of absorbance at 600 nm by means of a multi-detection microplate reader (Bio-Tek Synergy HT Microplate Reader, Bio-Tek Instruments, Winooski, VT, USA) and automatically recorded for each well every 20 min. The effect of NPs on bacterial growth and survival was evaluated by determining Range Concentrations (RC), Minimum Inhibitory Concentration (MIC), MIC50, and Minimum Bactericidal Concentration (MBC). MBC was determined by subculturing the total well volume (100 μL) to agar plates that do not contain the test agent. MBC was identified by determining the lowest concentration of NPs that reduced the viability of the initial bacterial inoculum by ≥99.9%.

### 2.3. Scanning Electron Microscopy

For observation under SEM, we adjusted the bacterial suspensions to 10^7^ cfu mL^–1^ in luria broth and added suspensions of the two variations of AgNPs to each bacterial sample to a final concentration of 75 μg/mL. After 5, 10, or 24 h of incubation, a 100 μL aliquot was placed on filter membranes with a 0.2 μm pore size. After rinsing with 0.1 M sodium cacodylate (SCB) buffer at pH 7.4, the samples were fixed with 2% paraformaldehyde and glutaraldehyde solution for 30 min. The samples were then washed twice with sodium cacodylate buffer and gradually dehydrated with increasing ethanol concentrations of 30%, 50%, 70%, 90%, and 100% (*v*/*v*) ethanol in water for 10 min, respectively. The samples were subsequently dried in a critical point drier (Baltec CPD 030), sputter-coated with a 10 nm thick layer of gold (Baltec SCD 050) and observed under a scanning electron microscope (JEOL JSM-6390 LV) (JEOL, Peabody, MA, USA) at an accelerating voltage of 25 kV.

### 2.4. Data Analysis

Measurements for each species were collected in triplicated 96-well plates, which were considered as different experimental blocks. OD measurements were modeled using Linear Mixed Effects Models (LMMs) in the statistical language R in two steps. First, to test for the existence of background effects, control samples (wells where pathogens were absent) were modeled using a LMM in which the dependent variable was the logOD. The fixed effects term included the moment of sampling (time) and concentration of the nanoparticle as categorical variables, as well as their interaction. The random term consisted of the triple measurements in each well (position effects), nested within each block. The function l_mer_ of the package l_me4_ in R was implemented [18]. Time of sampling and concentration of AgNPs had a significant effect on the OD reads, when pathogens were absent. Their interaction was significant, as well. Therefore, ordinary least square estimates of OD were extracted by the l_smeans_ function of the l_smeans_ package for each combination of time and concentration. These estimates were then subtracted from the experimental OD measures for the corresponding combinations of factors. The second step comprised the actual analysis, in which only samples containing pathogens were included. Experimental OD measures, resulted from subtraction of each background effect to the original measurements, were transformed as log(OD+1) and were submitted to a LMM as the dependent variable. The fixed and random components of the models were the same as in the first step. Each species was analyzed separately. Ordinary least square estimates of OD, and their corresponding standard errors, were extracted and plotted.

MIC for each species was identified as the minimum concentration of nanoparticle, for which the estimate of OD was not statistically different from the corresponding one at 0 h (Tukey pairwise comparisons were done by the implication of post hoc tests on the model estimates). MIC50 was interpolated from the graphs of OD vs. concentration [19]. Range concentrations were calculated as the minimum concentration of the nanoparticle, where the estimates of OD values were statistically lower than the estimates of OD values in the controls. Statistical significance was determined by Tukey post hoc comparisons on the corresponding LMM coefficient estimates.

## 3. Results

### 3.1. Characterization of NP1 and NP2

#### 3.1.1. UV-Vis Spectroscopy

**Figure 1:**
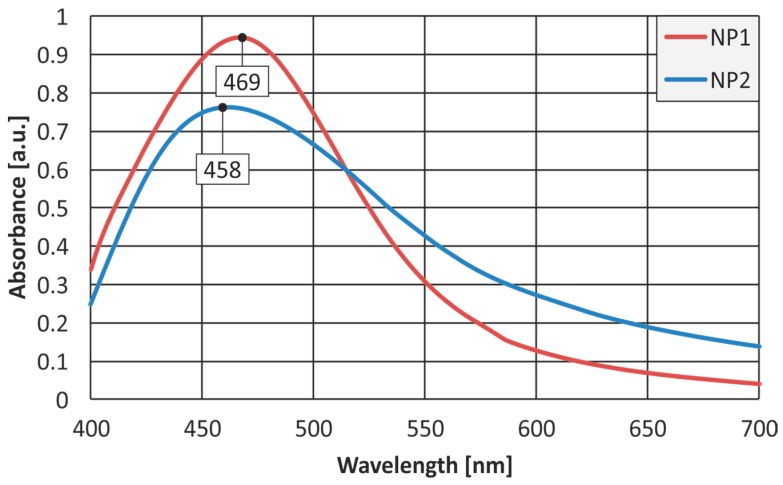
UV-Vis spectrometry results for both silver nanoparticles (AgNPs) variations silver nanoparticles variation 1 (NP1) and silver nanoparticles variation 1 (NP2).

Figure 1 shows the UV-Vis absorption spectra of the colloidal AgNPs for size variations NP1 and NP2. Before concluding about the optimum conditions to produce these variations, an experimental framework was performed to establish the process parameters for attaining mono-dispersity and long-term stability. In this context, variations by mixing different doses of LEA and silver nitrate solution at various temperatures and mixing durations were considered. The particles, although discrete, were predominantly coated with the organic component forming small groups, which makes them stable over long time periods. A higher and narrower absorption band peaked at around 469 nm was observed in the case of NP1, ascribed to the greater mean size of AgNPs produced when higher amounts of the leaf extract are incorporated. As for variation NP2, the absorption band was broadened and shifted to shorter wavelengths, at approximately 458 nm, due to the smaller mean size of the AgNPs at a much wider scatter. This will be discussed in the following section. Band peaks at higher wavelengths were also observed when higher LEA concentrations were applied for reducing Ag^+^ [20]. 

#### 3.1.2. Size and Shape of NPs Characterized by Dynamic Light Scattering and Transmission Electron Microscopy

**Figure 2:**
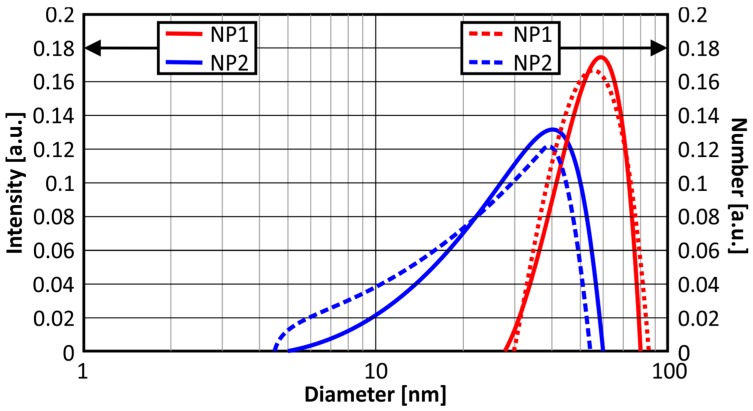
Dynamic light scattering results for both AgNPs variations NP1 and NP2.

Two different populations were measured through DLS for variations NP1 and NP2. The population of NP1 sample was characterized by a larger mean size, amounting to 58 nm, having a very narrow scatter (standard deviation: 18.4%). This behavior can be ascribed to the double quantity of leaf extract available for reducing AgNO_3_, yielding to a more abrupt nucleation and faster growth of the nanoparticles, while producing small agglomerates in some cases. The population of NP2 sample has a much smaller mean size of approximately 40 nm, however, it is characterized by a broader size scatter (standard deviation: 33.6%), especially to smaller sizes. In this sample, AgNPs with a size of 5 nm were measured, and most of them were mono-dispersed. The graphs presented in Figure 2 were created based on the most representative populations of variations NP1 and NP2, in terms of “intensity” (solid lines) and “number” (dashed lines). The differences in the plots between “intensity” and “number” are almost consistent with low polydispersion.

**Figure 3:**
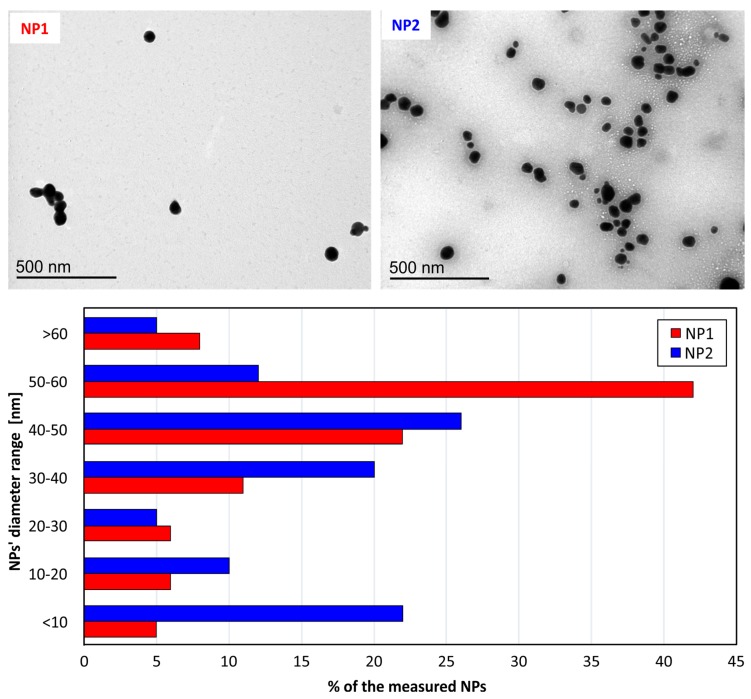
TEM images show the shape and size of both nanoparticles variations NP1 and NP2 with a concentration of 75 μg/mL. The graph at the bottom presents the size distribution of AgNPs.

Representative TEM images (Figure 3) show that both Ag nanoparticle variations, NP1 and NP2, are approximating a spherical shape. In the NP1 solution, we observe silver NPs with a diameter of mainly in the range of 50–60 nm, with a descending probability at smaller sizes. In the NP2 solution, we observe silver NPs with 40 nm diameter as the dominating size, whereas less NPs at smaller diameters tend to appear, but more NPs appear with diameter <10 nm. This evidence is in good agreement with the DLS measurements (i.e., mean NPs size for NP1 is 58 nm, and 40 nm for NP2) at large sizes, however, DLS seems to be unable to capture the high number of very small NPs (<10 nm).

#### 3.1.3. Surface Charge of NPs Measured by Zeta Potential

**Figure 4:**
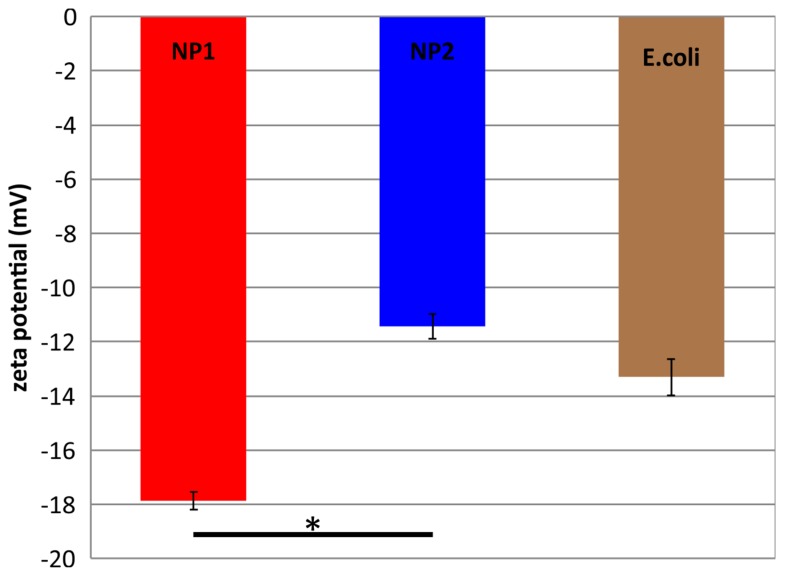
Zeta potential values of nanoparticle dispersion variations NP1 and NP2 with a concentration of 75 μg/mL. The difference between NP1 and NP2 is statistically significant, as shown by an asterisk (*p* < 0.05).

The measured zeta potential values of NP1 and NP2 dispersions were −18 and −11 mV, respectively (Figure 4). The *E. coli* dispersion has a z-potential value of −13 mV. The difference between the zeta potential values of NP1 and NP2 dispersions is statistically significant, as analyzed by the *t*-Test paired two samples for means.

### 3.2. Kinetics of Growth of Bacterial Pathogens under NP Antibiosis

**Figure 5:**
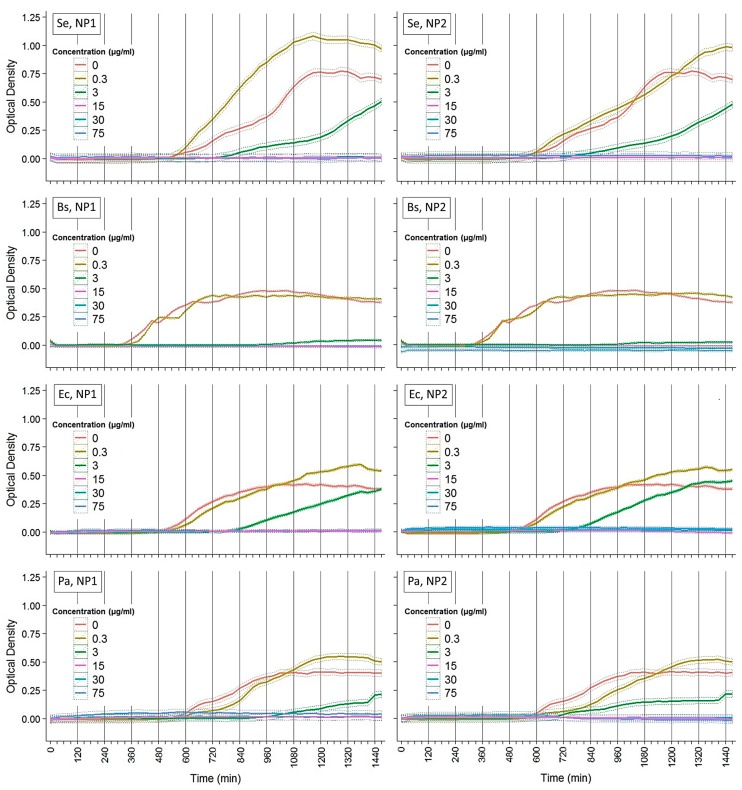
Kinetics of growth of bacterial pathogens under NP antibiosis. Optical densities were measured for 48 h at 37 °C using a multi-detection microplate reader at 600 nm and automatically recorded for each well every 20 min. Ordinary least square estimates and their standard errors calculated by LMMs (see methods) for three independent experiments of quadruplicate data sets are plotted here. Se: *S. epidermidis*; Bs: *B. subtilis* subsp. *subtilis*; Ec: *E. coli*; Pa: *P. aeruginosa*. Left panels: variation I NPs (NP1). Right panels: variation II NPs (NP2).

The growth kinetics of four bacterial species was monitored in 100 μL broth cultures, supplemented with a range of AgNP concentrations, which were incubated for 48 h (Figure 5). The lowest AgNP concentration (0.3 μg/mL) had no effect in bacterial growth in both cases of AgNP variations. Higher concentrations caused a growth delay or inhibition in all tested strains. Both variations of AgNPs at a concentration of 3 μg/mL caused a 5 h delay in the cases of *S. epidermidis* and *E. coli*. In the case of *P. aeruginosa*, growth delay differs (1.5 h) between AgNP1 (6.5 h) and AgNP2. *B. subtilis* was found to be more sensitive against the 3 μg/mL concentration of NP1 and NP2, which inhibited growth. In AgNP1,2 concentrations ≥15 μg/mL, bacterial growth was inhibited in all tested species. Thus, kinetic analysis suggests that growth of all species was affected even by low AgNPs concentrations, with *B. subtilis* being the most sensitive. Such effects were similar for both variations of AgNPs.

### 3.3. Dose Response Effect of NP1 and NP2 against Bacterial Pathogens

**Figure 6:**
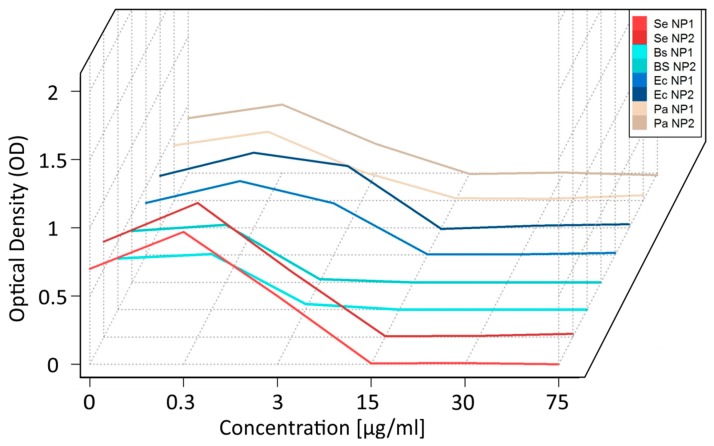
Comparative 3D representation of the dose response effect of nanoparticles variation I (NP1) and II (NP2) concentrations against bacterial pathogens that are named in Figure 5.

Figure 6 shows that bacterial sensitivity correlated to AgNPs concentration. *B. subtilis* was found to be the most sensitive, as an inhibition of growth was observed at 3 μg/mL, which was calculated as MIC of both AgNP variations (Table 1). Moreover, MIC coincided with MBC in the case of *B. subtilis*. MIC50 was slightly lower for AgNP2 compared to AgNP1 (1.8 or 2.01 μg/mL, respectively) (Table 1). A similar trend was observed in the case of the other gram-positive species, *S. epidermidis*; growth was inhibited at 15 μg/mL (MIC) due to a bactericidal effect (MBC = 15 μg/mL). Again, MIC50 was slightly lower for AgNP2 than AgNP1 (6.3 or 6.6 μg/mL, respectively). In the case of the gram-negative *E. coli* or *P. aeruginosa*, MIC and MBC were also calculated at 15 μg/mL. MIC50 was found slightly lower for AgNP1 compared to AgNP2 (9 or 9.9, respectively) in the case of *E. coli*, but was the same (3.6) for both variations in the case of *P. aeruginosa*. The fact that range concentrations coincide with MICs and MBC for each species tested suggests that: (a) NPs were highly toxic to bacteria; and (b) there is a certain threshold in the number of AgNPs around the bacterial cell above which cells collapse and die. 

**Figure 7:**
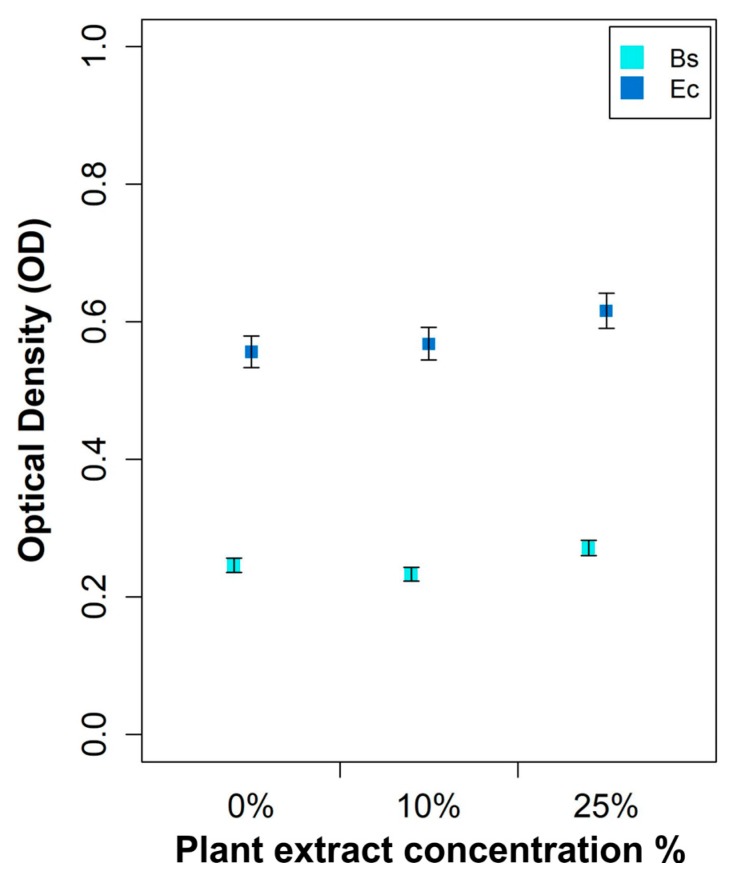
Representation of the dose response effect of *Arbutus unedo* leaf extract at two different concentrations against *B. subtilis* (Bs) and *E. coli* (Ec). No significant differences in optical density were detected within each species with respect to the null concentration.

To ensure that low MIC and MBC levels found were not due to antimicrobial properties of the plant extract used to produce AgNPs, positive controls were also tested. Specifically, bacterial growth was assessed for 24 h in wells containing a final concentration of 10% or 25% of the plant extract in the absence of AgNPs (blank formulation). Growth was found to be like that in the case of the control (0%: medium only) (see Figure 7).

### 3.4. Morphology of E. coli Cells under NP1 and NP2 Antibiosis

**Figure 8:**
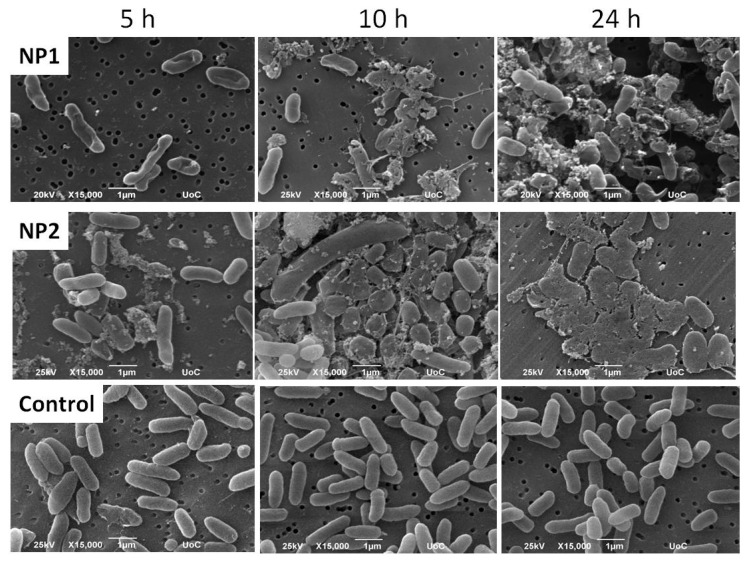
Representative scanning electron microscopy images showing the induction of 75 μg/mL nanoparticles on *E. coli* for 5, 10, 24 h. Upper panel: variation I NPs (NP1); Middle panel variation II NPs (NP2); Lower panel: Control (0% NPs, *E. coli* only). Magnification is 15,000× and scale bar represents 1 μm.

We observe the morphological change of the *E. coli* cell membranes under induction with 75 μg/mL of AgNPs for 5, 10, and 24 h under SEM, and compare them to the non-treated bacteria (Figure 8). Representative SEM images show that NP1 form aggregates around the bacteria after 5 h, and lead to an alteration of the physiological rod-shaped bacteria (Figure 8, left panels). Longer interaction times (10 h) with NP1 show a more pronounced bacterial shape change resulting in smaller, round bacteria with damaged membranes (Figure 8, upper central panel), an effect which develops into an extended membrane disruption after 24 h (Figure 8, upper right panel). Notably, the NP2 significantly affect the bacterial shape after 5 h of induction (Figure 8 middle left panel), leading to a shrinkage of the bacterial membrane and introducing holes, which are also observed after 10 h with a strong membrane damage, which increases dramatically after 24 h (Figure 8, middle central or right panels, respectively). 

## 4. Discussion

Two variations of silver nanoparticles, named as NP1 and NP2, were synthesized by an eco-friendly route, exploiting the high reduction power that leaf extracts offer [15,22]. Through this, a minimum use of chemicals (just AgNO_3_) is required, minimizing the environmental impact that conventional silver nanoparticles possess. The major challenge posed by bio-synthesis methods is the stability and reproducibility of the process. However, when this is mastered, low-cost and ultra-stable nanoparticles can be produced, given that there is no need for high pressure, temperature or energy in this green fabrication process. The high stability of NP1 and NP2 shown in this study is due to the organic coating formed around the nanoparticles [15]. Silver nanoparticles stabilized with poly(*N*-vinylpyrrolidone) with diameters of 50 nm have been reported to have a zeta potential of −17 mV, whereas citrate stabilized AgNPs had −30 to −45 mV, depending on silver concentration [23]. Our results are in good agreement with a previous report, indicating a nanoparticle mean size of 50 nm and zeta potential values of −18 and −11 mV for NP1 and NP2. Although significant differences of charge were not observed between NP1 and NP2, the slightly lower charge of NP2 could be attributed to the lower quantity of LEA, yielding to a thinner coating on the surface of NPs.

Both variations of AgNPs were effective in vitro against the tested species, with *B. subtilis* being the most sensitive, comparable with efficacies reported on previous studies [24]. The overall antibacterial efficacy of AgNPs developed in this study against the four bacterial species yields MICs and MBCs that are comparable or in some cases, lower than those reported by other groups [24,25]. However, a direct comparison between data in the literature and our results was not possible, given that starting compositions, capping agents and the resulting size of NPs differ in some cases significantly. The better activity against gram-negative species that has been reported by these groups was not observed in this study, suggesting that AgNPs activity was independent of the cell wall structure. In fact, scanning electron microscopy in time samples of liquid cultures of *E. coli*, the least sensitive among the species tested, and in the presence of AgNP1, revealed that co-incubation of 5 h disrupted the integrity of the bacterial membrane and cell wall. It is known that AgNPs’ action largely relies on releasing silver ions that damage membranes and affect several biochemical processes. However, they especially interact with thiol and amino groups of proteins and induce the release of reactive oxygen species (ROS), forming free radicals with strong bactericidal action [2,26].

Most of the factors that are known to modify the antibacterial effect of AgNPs, such as size, shape, stability, and concentration [1] were evaluated in this study. The size-dependent activity of AgNPs attributed to a relative increase of the surface area to volume ratio of nanoparticles and the contact surface area has been reported by other research groups [27,28]. The larger size of NP1 compared to NP2 shown in our work, similar to nanoparticles synthesized by other groups [3,4,25], did not seem to have a significant effect on the overall efficacy. However, a slight difference in the bactericidal activity in favor of AgNP2 was observed in SEM figures, suggesting a slight elevation in bactericidal activity that could not be observed in the kinetics of growth (inhibition was the same), or impact MBC calculations.

It should be noted that some agglomeration of nanoparticles was observed after incubation, which was resolved by mild sonication. Such an agglomeration was more evident after co-incubation of AgNPs with the pathogens, due to the additional binding of nanoparticles to cell debris. Since nanoparticles are known to absorb biomolecules and agglomerate in the biological mediums that are usually tested [29], their efficacy might be different in dry surfaces, which could be the subject of another study. 

Our results showed a strong bactericidal effect of synthesized AgNPs that can be translated into a biomedical application of great potential. This could address the shortage of new antimicrobials against the emerging antimicrobial resistant microorganisms, which are mostly gram-negative bacteria. In addition, the green synthesis offers a one-step fabrication and stabilization of the nanoparticles, since the plant extract reduces Ag^+^ to Ag^0^, and the material constituents act as capping agents to the system, simultaneously adding biocompatible functionalities into these NPs for enhancing further biological interactions [26]. The low toxicity of such NPs in mammalians reduces environmental risks, such as acute toxic effects or bioaccumulation [30].

## 5. Conclusions 

In this study, we report on the production of silver nanoparticles (AgNPs) in a cost-effective and environmentally friendly way using extract of the plant *Arbutus unedo* (LEA) to reduce silver ions. As the size control and the stability of nanoparticles remain challenging issues, we produced two size variations of silver AgNPs by varying the LEA concentration. Both variations were evaluated for their antibacterial efficacy against the gram-negative species *E. coli* and *P. aeruginosa*, and the gram-positive species *B. subtilis* and *S. epidermidis*. All four bacterial strains were tested sensitively against both variations of AgNPs due to bacterial membrane disruption as indicated by microscopy. Although the nanoparticles’ size between the two variations was different, their antibacterial effect was similar in all investigated strains.

## Figures and Tables

**Table 1:** Summary of the broth microdilution (microtiter) efficacy tests of NP concentrations against four bacterial strains. Plot smoothing of the modeled bacterial responses after 24 or 48 h of incubation by local polynomial regression and projection to the concentration axis for the 50% of the initial OD values were implemented to interpolate MIC50 values. The loess function in programming language R [21] was applied for calculations.

Species	MIC (μg/mL)		MIC50 (μg/mL)		Range Concentrations (≥) (μg/mL)				MBC (μg/mL)	
	NP1	NP2	NP1	NP2	NP1 12 h	NP2 12 h	NP1 24 h	NP2 24 h	NP1	NP2
*S. epidermidis*	15	15	6.6	6.3	-	-	15	15	15	15
*B. subtilis*	3	3	2	1.8	3	3	3	3	3	3
*E. coli*	15	15	9	9.9	3	3	15	15	15	15
*P. aeruginosa*	15	15	3.6	3.6	-	-	15	15	15	15
